# Exploring internal representations of self-supervised networks: few-shot learning abilities and comparison with human semantics and recognition of objects

**DOI:** 10.3389/fncom.2025.1613291

**Published:** 2025-11-21

**Authors:** Asaki Kataoka, Yoshihiro Nagano, Masafumi Oizumi

**Affiliations:** 1 Graduate School of Arts and Sciences, The University of Tokyo, Meguro, Japan; 2 Graduate School of Informatics, Kyoto University, Sakyo, Japan

**Keywords:** contrastive learning, few-shot learning, human semantics, human recognition, similarity, self-supervised learning

## Abstract

Recent advances in self-supervised learning have attracted significant attention from both machine learning and neuroscience. This is primarily because self-supervised methods do not require annotated supervisory information, making them applicable to training artificial networks without relying on large amounts of curated data, and potentially offering insights into how the brain adapts to its environment in an unsupervised manner. Although several previous studies have elucidated the correspondence between neural representations in deep convolutional neural networks (DCNNs) and biological systems, the extent to which unsupervised or self-supervised learning can explain the human-like acquisition of categorically structured information remains less explored. In this study, we investigate the correspondence between the internal representations of DCNNs trained using a self-supervised contrastive learning algorithm and human semantics and recognition. To this end, we employ a few-shot learning evaluation procedure, which measures the ability of DCNNs to recognize novel concepts from limited exposure, to examine the inter-categorical structure of the learned representations. Two comparative approaches are used to relate the few-shot learning outcomes to human semantics and recognition, with results suggesting that the representations acquired through contrastive learning are well aligned with human cognition. These findings underscore the potential of self-supervised contrastive learning frameworks to model learning mechanisms similar to those of the human brain, particularly in scenarios where explicit supervision is unavailable, such as in human infants prior to language acquisition.

## 1 Introduction

Self-supervised learning has recently gained significant attention from both the machine learning and neuroscience communities. Unlike supervised learning, which requires explicit task-specific labels, self-supervised learning relies on inherent structures within the data itself and does not require manual supervision. This property makes it particularly advantageous in machine learning, enabling models to be trained on vast amounts of uncurated (unlabeled) data. Recent studies have demonstrated the effectiveness of self-supervised learning as a powerful method for representation learning (Arora et al., 2019; Medina et al., 2020; Chen and He, 2020; Newell and Deng, 2020; Ericsson et al., 2022; Nozawa and Sato, 2021; Shi et al., 2021; Wang et al., 2021; Bao et al., 2022; Zhu et al., 2022; Hu et al., 2024).

In neuroscience, it is equally important to investigate the characteristics of neural representations that emerge from self-supervised learning, as this can provide insights into learning mechanisms in the brain. Given that self-supervised learning does not require labeled input, it offers a plausible framework for brain-like learning. In particular, since language is considered a major source of supervision in humans (Knudsen, 1994; Glaser et al., 2019; Loewenstein et al., 2021), self-supervised learning may play a central role in the brains of human infants before language acquisition, as well as in non-linguistic animals. When applied to the study of neural learning and information representation, self-supervised learning may help explain empirical findings showing that prelinguistic infants exhibit cognitive abilities–such as categorical representation—similar to those of adults (Carey and Bartlett, 1978; Quinn et al., 1993; Behl-Chadha, 1996; Freedman et al., 2001; Smith et al., 2002; Yang et al., 2016).

Deep convolutional neural networks (DCNNs) have frequently been used as computational models of neural circuits to study such neural representations. Earlier studies have shown that DCNNs trained with supervised learning exhibit representational similarities to the visual systems of humans and animals (Lecun et al., 1998; Kriegeskorte et al., 2008; Jarrett et al., 2009; Krizhevsky et al., 2012; Yamins et al., 2013, 2014; Khaligh-Razavi and Kriegeskorte, 2014; Majaj et al., 2015; Yamins and DiCarlo, 2016; Rafegas and Vanrell, 2018; Rajalingham et al., 2018; Hebart et al., 2020; Marques et al., 2021; Kawakita et al., 2024). Building on this foundation, recent work has demonstrated that DCNNs trained using self-supervised algorithms also show representational similarities to biological visual systems (Bakhtiari et al., 2021; Zhuang et al., 2021; Nayebi et al., 2021; Konkle and Alvarez, 2021; Cadena et al., 2019; Konkle and Alvarez, 2022; Millet et al., 2022; Prince et al., 2024), further supporting their plausibility as models of the visual system.

In this study, we investigate the internal representations of deep convolutional neural networks (DCNNs) through the lens of inter-category relationship structures, as revealed by *few-shot learning* performance. Few-shot learning refers to the ability to recognize novel, previously unseen categories using only a limited number of examples. While prior studies have highlighted the similarity between DCNN representations and those of humans and animals, the structure of category-level representations has not been thoroughly explored. A recent study (Sorscher et al., 2022) evaluated the few-shot learning capabilities of DCNNs trained with both supervised and self-supervised methods. Building upon this work, we compare the category structures revealed through few-shot learning in DCNNs with human semantic organization and recognition performance, aiming to further clarify the nature of internal representations learned without explicit supervision.

In this paper, we pursue three objectives: (i) to confirm that DCNNs trained with self-supervised learning can perform few-shot learning accurately, and (ii) to investigate their internal representations by comparing them to human semantic organization and (iii) human recognition performance. The few-shot learning ability (i) is evaluated based on the linear separability of categories within the neural representation space. From this evaluation, we derive category-wise confusion matrices for each trained DCNN. These matrices are then used to analyze the inter-category structure of the representations and to compare them to (ii) human semantic structures and (iii) human recognition patterns. While prior studies have investigated self-supervised DCNNs (Medina et al., 2020; Sorscher et al., 2022; Lu et al., 2022), our novel contribution lies in the comparative analyses involving human-level semantics and recognition–specifically, objectives (ii) and (iii).

Our experimental results indicate that the internal representations arising from self-supervised learning in DCNNs closely resemble human semantic structures and recognition patterns. These findings suggest that inter-category structures similar to those found in human cognition can emerge even before the application of explicit supervision, thereby supporting both the biological plausibility and practical utility of self-supervised learning in brain-like systems.

## 2 Materials and methods

### 2.1 Overall evaluation procedure

**Figure 1:**
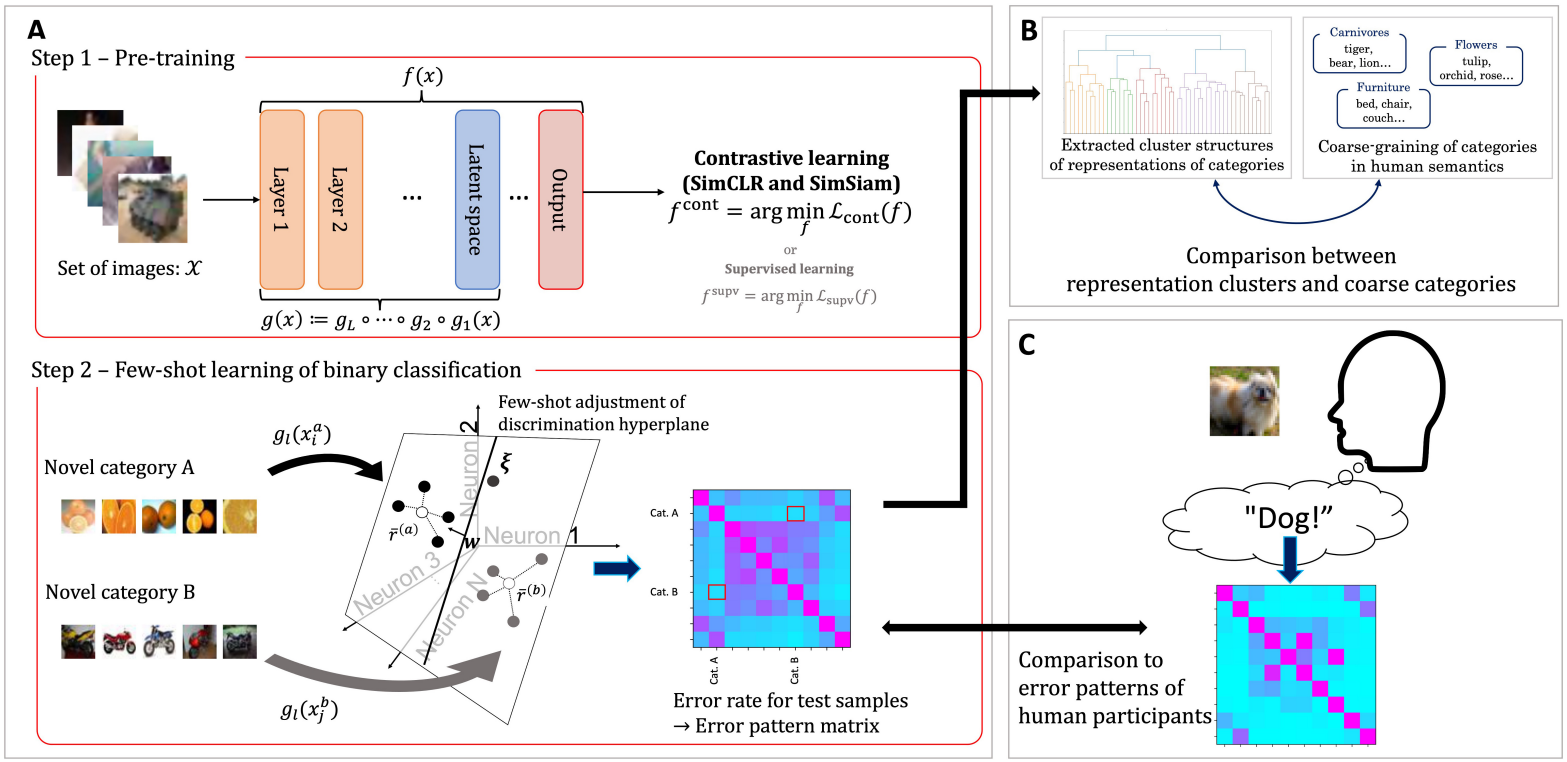
Schematic illustration of the experimental procedure in this study. **(A)** A two-step methodology for evaluating the few-shot learning performance of DCNNs. In Step 1 (pre-training), the network is trained using self-supervised contrastive learning. In Step 2, pairwise few-shot classification is performed, and performance is assessed using error pattern matrices, where each cell represents the classification error rate between a pair of novel categories. **(B)** Clusters of object categories derived from the error pattern matrices are compared to coarse-grained category groupings based on human semantic relationships. **(C)** Similarity is evaluated between the error pattern matrices of the DCNNs and the confusion matrix obtained from human participants performing an object classification task.

This study evaluates the extent to which the internal representations of visual objects in DCNNs trained by self-supervised learning framework resemble human perceptions of them. In particular, we focus on the categories of objects. We quantitatively evaluate correspondence between the inter-categorical relationship structure within learned internal representations of the DCNNs and human semantics and recognition. The overall evaluation procedure involves pre-training of the DCNNs (Figure 1A, top), evaluation of their few-shot learning ability (Figure 1A, bottom), and quantitative evaluation of the correspondences (Figures 1B, C).

In the first step (Figure 1A, top), we pre-train a DCNN with a self-supervised contrastive learning (Jaiswal et al., 2020; Kumar et al., 2022). The objective function utilized for the contrastive learning framework does not require explicit supervision signals over object categories. To assess the impact of the absence of supervision, we also train a separate DCNN using a supervised object classification task as a baseline for comparison. The evaluation of few-shot learning performance is then conducted in the next step.

In the second step (Figure 1A, bottom), we evaluate the few-shot learning performance of the DCNNs, specifically the linear separability of internal representations into the target categories. During this evaluation, the synaptic weights of the DCNNs are kept frozen. The networks are presented with images from novel object categories that were not included during pre-training. A few exemplar images from each category are used to compute “prototype” representations, and the remaining samples are classified based on their similarity to these prototypes. The classification results are summarized in confusion matrices, which we refer to hereafter as error pattern matrices.

After obtaining the error pattern matrices, we perform analyses to examine how closely the internal representation structures of the networks resemble those of humans. To evaluate the similarity of these structures in detail, we adopt the following two approaches. The first approach (Figure 1B) evaluates how the grouping of object categories in the internal representations of DCNNs aligns with human semantic organization. Using the error pattern matrices obtained from the few-shot learning task, we perform hierarchical clustering to identify clusters of categories that are represented closely together. We then quantify the extent to which the categories within each cluster correspond to predefined coarse-grained object categories. In the second approach (Figure 1C), we quantitatively evaluate the similarity of error patterns between human participants and DCNNs in object classification tasks. Specifically, we use a dataset of object images and a confusion matrix derived from human participants performing multi-label classification of these images (see Section 2.2.2). We then compare this human confusion matrix with confusion matrices obtained from the multi-class few-shot learning evaluations of the networks.

### 2.2 Dataset

#### 2.2.1 Image dataset: CIFAR-100

In the pre-training phase and evaluation of pairwise few-shot learning performance, we utilized the CIFAR-100 dataset (Krizhevsky, 2019). This dataset consists of 60,000 colored images of objects each with a resolution of 32 x 32 pixels. In this dataset, each image has two different labels to annotate which category the object in the image belongs to, namely *fine category* and *coarse category*. The number of fine categories defined in the dataset is 10, and each fine category belongs to one of 20 coarse categories. The number of included fine categories in each coarse category is 5. For instance, the coarse category *large carnivores* include *bear, leopard, tiger, wolf*, and *lion*. The number of image samples in each category is equal; each coarse category contains 3, 000 images, and each fine category contains 600 images.

#### 2.2.2 Human visual classification task dataset: CIFAR-10H

The dataset used to evaluate the similarity of error patterns between the DCNNs and human participants is CIFAR-10H (Battleday et al., 2020). This dataset was collected in a behavioral experiment in which 2,750 human participants classified images from the well-known CIFAR-10 (Krizhevsky, 2019) dataset into 10 object categories. Participants were instructed to select the object category for each image as quickly as possible after its presentation. Although humans are expected to perform this task more accurately than machines, some misclassification errors were inevitably observed.

The dataset provides the results of the behavioral experiment, with the number of human participants classifying each image into each of the 10 categories. By averaging the histograms of these classifications within each ground-truth object category, we generate a misclassification pattern histogram for that category. These misclassification histograms were then arranged to form a confusion matrix representing behavior of human participants. In particular, given the misclassification histogram for each image sample, averaging the histograms over all images belonging to a certain object category yields a confusion histogram of a certain object category into others. The confusion matrix is obtained by stacking the confusion histogram for different categories. Assuming that this confusion matrix reflects the similarity relationship between object categories in human recognition, we later compare it with the error pattern matrices obtained from the DCNNs to assess the correspondence between the DCNNs' internal representations and those of humans (see Section 3.3 for the results).

#### 2.2.3 Categories in the datasets

To evaluate few-shot novel category discrimination, we first define “known categories” as those present during the pre-training phase, and “novel categories” as those absent in it. For the CIFAR-100 dataset, we randomly divided the 20 coarse categories into two subsets (10 coarse categories for each) and then assigned the corresponding fine categories based on this division. Pre-training (either contrastive or supervised) was conducted using only input images belonging to the known categories. This ensures that none of the novel categories used during the evaluation of few-shot discrimination were encountered by the network during pre-training.

In addition, we conducted a separate few-shot discrimination test to generate error pattern matrices for comparison with human semantics and recognition. For this purpose, CIFAR-100 categories could not be used, as the human confusion matrices were constructed based on the category definitions of the CIFAR-10 dataset. Importantly, the category and image sets of CIFAR-10 are completely disjoint from those of CIFAR-100. This guarantees that the CIFAR-10 images also represent novel categories from the perspective of the pre-trained network.

### 2.3 Pre-training

In the first step, we train DCNNs f:X→Y using self-supervised contrastive learning, and also train a separate model with supervised object classification as a baseline. Both models share the same encoder architecture g:X→Z, which maps an input image to a common latent space. From this latent space, distinct projection heads proj:Z→Y are used, such that *f*: = proj∘*g*. Note that the projection heads differ between the two models, and consequently, the dimensionality of Y is not the same across them. Since we aim to evaluate whether the learned representations apply to discrimination between novel object categories which are unseen in the pre-training, both networks are trained on the “known” half of CIFAR-100 dataset (see Section 2.2.3).

#### 2.3.1 Self-supervised contrastive learning

In this work, we adopt *self-supervised contrastive learning* as a representative framework of learning rules that do not rely on explicit supervision signals. In contrastive learning, a network is trained such that the internal representations of *semantically similar* inputs (positive pairs) are brought closer together, while those of dissimilar inputs (negative pairs) are pushed apart in the network's latent space. Specifically, we use SimCLR (Chen et al., 2020) as a standard contrastive learning algorithm. We also include SimSiam (Chen and He, 2020) as an additional contrastive method to evaluate the robustness of our findings (see Supplementary material and Supplementary Figure S1).

**Figure 2:**
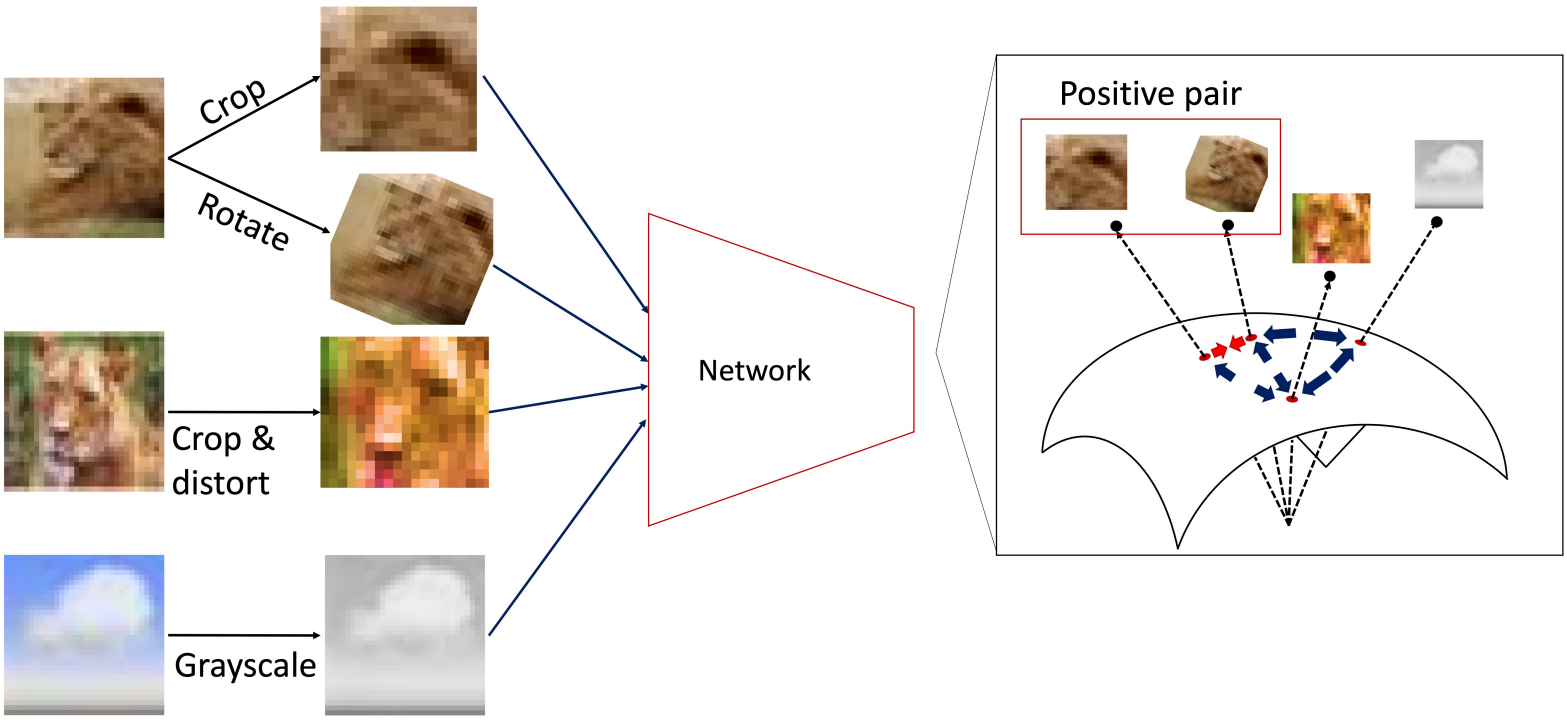
Schematic illustration of SimCLR contrastive learning. During SimCLR training, the DCNN is provided with a large number of image inputs, each generated from an original image by applying random augmentations such as cropping, rotation, or color distortion. In SimCLR, the network is trained so that internal representations of augmented views from the same original image (i.e., *positive pairs*) are mapped close together in the latent space, while representations of all other combinations (*negative pairs*) are pushed farther apart.

SimCLR applies a randomly selected combination of augmentations to each input image and treats two differently augmented views of the same image as a positive pair during training. Figure 2 provides a schematic illustration of instances of such augmentations, including random cropping, rotation, color distortion, and grayscaling. Table 1 outlines the specific augmentation rules and parameters we used.

**Table 1:** Augmentation rules and corresponding parameters.

**Name of augmentation**	**Parameters**
Random cropping	Scale: [0.08, 1.0]
	Ratio: [0.75, 1.25]
Horizontal flipping	Probability: 0.5
Color jittering	Strength: 0.5
	Probability of grayscaling: 0.2

Suppose an augmentation function a∈A is sampled from a probability distribution $\rho_{\text{aug}}\in \mathbb{P}\left(A\right)$, and an input image x∈X is sampled from another distribution $\rho_{\text{im}}\in \mathbb{P}\left(X\right)$. Here, a:X→X represents a single composition of randomly applied augmentations listed in Table 1. The neural network $f:X\to {\mathbb{R}}^{d}$ is trained on those augmented input samples. The informative neighborhood contrastive estimation (*InfoNCE*) loss function for SimCLR is defined as


$$\begin{array}{l} L_{\text{CLR}}\left(f\right)\\ : =-{𝔼}_{x,\left\{x_{k}^{-}\right\}\sim \rho_{\text{im}}^{K+1},\left\{a_{k}\right\}\sim \rho_{\text{aug}}^{K+2}}\\ \left[l_{\text{CLR}}\left(a_{K+1}\left(x\right),a_{K+2}\left(x\right),{\left\{a_{k}\left(x_{k}^{-}\right)\right\}}_{k=1}^{K};f\right)\right], \end{array}$$ (1)

$$\begin{array}{l} l_{\text{CLR}}\left(\tilde{x},{\tilde{x}}^{+},{\left\{{\tilde{x}}_{k}^{-}\right\}}_{k=1}^{K};f\right)\\ : =\log\frac{\exp\left(\hat{f}\left(\tilde{x}\right)\cdot \hat{f}\left({\tilde{x}}^{+}\right)\right)}{\exp\left(\hat{f}\left(\tilde{x}\right)\cdot \hat{f}\left({\tilde{x}}^{+}\right)\right)+\sum_{k=1}^{K}\exp\left(\hat{f}\left(\tilde{x}\right)\cdot \hat{f}\left({\tilde{x}}_{k}^{-}\right)\right)}, \end{array}$$ (2)

where ${\left\{x_{k}\right\}}_{k=1}^{K}\sim \rho^{K}$ indicates that {*x*_1_, …, *x*_*K*_} are independently sampled from the same distribution ρ, and $\hat{f}\left(\cdot \right):=f\left(\cdot \right)/\|f\left(\cdot \right)\|$ denotes the normalized internal representation. Here, $\tilde{x}$, ${\tilde{x}}^{+}$, and ${\left\{{\tilde{x}}_{k}^{-}\right\}}_{k=1}^{K}$ represent the anchor, the positive, and the negative samples, respectively. As shown in Equation 1, positive pairs are generated by applying different random augmentations to the same image. In Equation 2, the symbol · denotes the inner product between two vectors. Minimizing Equation 2 can be interpreted as maximizing similarity of representations within the positive pair $\left(\tilde{x},{\tilde{x}}^{+}\right)$, while minimizing the similarity of them to the negative samples ${\left\{{\tilde{x}}_{k}^{-}\right\}}_{k=1}^{K}$. Note that computing the exact expectation in Equation 1 is computationally infeasible due to multiple integrals over continuous random augmentations. Therefore, we approximate it using the empirical mean over a minibatch.

#### 2.3.2 Supervised object classification learning

For comparison with the network trained using the contrastive learning algorithm, we also consider supervised object classification learning. This approach requires explicit supervision signals that specify the object category to which each input image belongs. The network is trained such that its output, interpretable as estimated probabilities over the object categories, closely matches the ground-truth labels provided by the supervision signals.

The loss function for the network $f:X\to {\mathbb{R}}^{\left|C\right|}$ is defined as


$$\begin{array}{l} L_{\text{supv}}\left(f\right)=-{𝔼}_{\left(x,y\right)\in D}\underset{c\in C}{\sum }y_{c}\log{\text{softmax}}_{c}\left(f\left(x\right)\right), \end{array}$$ (3)

where $D:={\left\{\left(x_{i},y_{i}\right)\right\}}_{i=1}^{N}$ denotes the dataset, C is a pre-defined set of object categories in the training −> data, and *f* indicates the network being trained. The subscript *c* denotes the index of a |C|-dimensional vector. *y* is an element of a probability simplex $\Delta^{\left|C\right|}$, and is typically a *one-hot* vector, *i.e*., $\left\{y\in {\left\{0,1\right\}}^{\left|C\right|}|\underset{c\in C}{\sum }y_{c}=1\right\}\subset \Delta^{\left|C\right|}$. The softmax function, which outputs the probability that *x* belongs to category *c*, is defined as


$$\begin{array}{l} {\text{softmax}}_{c}\left(f\left(x\right)\right)=\frac{\exp\left(f_{c}\left(x\right)\right)}{\sum_{c^{\prime }\in C}\exp\left(f_{c^{\prime }}\left(x\right)\right)}. \end{array}$$ (4)

Optimization is performed using gradient descent with error back-propagation (Rumelhart et al., 1986). To improve robustness to noise, we also applied random augmentations to the input images. The set of augmentations was identical to that used in the self-supervised contrastive learning setting (see Table 1). As in the contrastive learning case, the expectation in Equation 3 is approximated by empirical average over minibatches due to computational constraints.

### 2.4 Few-shot learning

One of the main goals of this study is to investigate whether DCNNs trained with self-supervised learning algorithms can accurately perform few-shot classification of novel object categories. To this end, we follow the approach (Sorscher et al., 2022) and formalize few-shot learning as the linear separability of internal representations of the novel categories.

The details of the few-shot learning evaluation procedure are as follows. Let {*c*_1_, ..., *c*_*n*_} be the novel fine object categories that have not been used for the pre-training phase. From each category *c*_*j*_, we randomly sample *m* training examples (with *m* = 10 in this study), denoted as $x_{i}^{\left(c_{j}\right)}\left(i=1,...,m\right)$. During pre-training, the network output is computed as *f*(*x*) = (proj∘*g*)(*x*), where *g* is the encoder and proj is the projection head. In contrast, for few-shot evaluation, we extract representations from the *l*-th layer of the encoder, denoted *r* = *g*_*l*_(*x*), where *g* = *g*_*L*_∘...∘*g*_1_. For each novel category *c*_*j*_, we compute a “prototype” representation by averaging the internal representations of its training samples:


$$\begin{array}{l} {\bar{r}}^{\left(c_{j}\right)}:=m^{-1}\overset{m}{\underset{i=1}{\sum }}r_{i}^{\left(c_{j}\right)}. \end{array}$$ (5)

Given these prototypes, a test sample ξ is classified into the category $\tilde{c}$ defined by


$$\begin{array}{l} \tilde{c}=\underset{j}{\arg\;\min}{\left\|{\bar{r}}^{\left(c_{j}\right)}-\xi \right\|}_{2}. \end{array}$$ (6)

This procedure evaluates whether the DCNN organizes internal representations such that inputs from the same category are embedded closely, while those from different categories are well separated.

In particular, when *n* = 2, this procedure admits an alternative geometric interpretation. Given the prototypes ${\bar{r}}^{\left(c_{1}\right)}$ and ${\bar{r}}^{\left(c_{2}\right)}$ for the two categories *c*_1_ and *c*_2_, we can define a linear decision boundary as follows:


$$\begin{array}{l} w={\bar{r}}^{\left(c_{1}\right)}-{\bar{r}}^{\left(c_{2}\right)}, \end{array}$$ (7)

$$\begin{array}{l} \beta =\frac{1}{2}w\cdot \left({\bar{r}}^{\left(c_{1}\right)}+{\bar{r}}^{\left(c_{2}\right)}\right). \end{array}$$ (8)

For a test sample with representation ξ, the predicted category is *c*_1_ if


$$\begin{array}{l} h=w\cdot \xi -\beta \end{array}$$ (9)

is greater than zero, and *c*_2_ otherwise. Since this yields exactly the same classification result as the prototype-based method described in the previous paragraph for *n* = 2, this procedure can equivalently be interpreted as constructing a linear discrimination hyperplane between a pair of novel object categories and evaluating its generalizability to test samples.

Hereafter, we refer to the case of *n* = 2 as *pairwise few-shot learning*, and the case of *n* > 2 *multi-class few-shot learning*. In pair-wise few-shot learning evaluation, a confusion matrix is generated by iteratively conducting evaluations for all possible pairs of novel categories, whereas is multi-class few-shot learning, a confusion matrix is constructed from a single evaluation involving all novel categories as candidate object classes. The results of the pairwise few-shot learning evaluation are presented in Section 3.1. The multi-class few-shot learning evaluation is used to compare the error patterns of DCNNs with those of human participants, and the corresponding results are shown in Section 3.3.

Practically, we also add a several conditions to the evaluation procedure. First, this evaluation is done independently at each layer with different dimensionality of internal representation and different representational nature. Hence, the results can vary between different layers. Second, since the result varies for different random choices of the training samples and test samples, we show averaged value over different choices for each element of the resulting error pattern matrices. This can lead to robust and general tendency of how the trained models differentiate between novel object categories. Third, in order to guarantee that the categories and samples used for this evaluation is unseen during the pre-training step, we use the novel subset of CIFAR-100 dataset in this evaluation (see Section 2.2.3).

### 2.5 Comparison between the models and human semantics and recognition

The primary objective of this study is to evaluate the similarity between the inter-categorical structure of internal representations in DCNNs and that of human semantics and recognition. To this end, we employed two complementary methods of evaluation. The following subsections describe these methods in detail.

#### 2.5.1 Clustering-based evaluation: comparison with human-defined categorical aggregation

**Figure 3:**
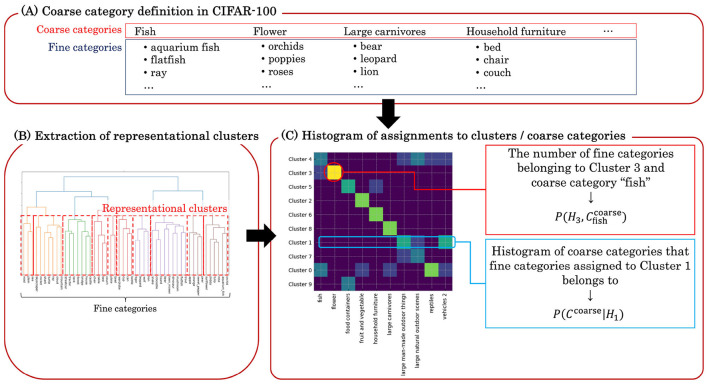
Schematic illustration of clustering-based evaluation procedure. **(A)** The evaluation adopts the coarse category definitions provided by the CIFAR-100 dataset. The dataset for evaluation includes 10 coarse categories, each containing 5 fine categories. **(B)** An example of hierarchical clustering of fine categories based on the error pattern matrix obtained from the pairwise few-shot learning evaluation. Given that the error rates can be interpreted as the representational dissimilarities, hierarchical clustering merges the fine categories at different levels of dissimilarities. We find the dissimilarity threshold at which the number of highest-level clusters is 10, matching the number of coarse categories provided by CIFAR-100. **(C)** An example of a joint histogram of which representational clusters / coarse categories a fine category is assigned to. Each element represents the number or fraction of fine categories assigned to a certain pair of a cluster and a coarse category.

This analysis examines whether the emergent similarity structure among object categories in the learned representations of DCNNs reflects the categorical organization of human semantics. To investigate this, we use the coarse categories from the CIFAR-100 dataset, which defines two levels of object categories (Section 2.2.1 and Figure 3A): fine (*e.g*., wolf, lion, leopard, bear, and tiger) and coarse (*e.g*., large carnivores). Focusing on the 10 coarse categories within a novel subset of the dataset, our analysis evaluates the extent to which these pre-defined fine-to-coarse inclusion relationships are mirrored by the aggregations that emerge within the similarity structure of the DCNN's internal representations.

To extract these similarity-based groupings, we perform hierarchical clustering on the error pattern matrices derived from a pairwise few-shot learning evaluation (Figure 3B). We term the resulting groups representational clusters. Since the few-shot evaluation is conducted at the fine-category level, the error pattern matrix can be interpreted as a dissimilarity matrix between fine categories. Hierarchical clustering progressively merges the most similar fine categories or lower-level clusters, producing a dendrogram that illustrates the groupings at various levels of dissimilarity. To align our analysis with the semantic structure in the dataset, we cut the dendrogram at the level that yields 10 top-level clusters, matching the number of coarse categories.

We then quantify the consistency between the predefined coarse categories and the representational clusters using mutual information. The goal is to evaluate the similarity between the two partitioning schemes of the fine categories, or in other words, to measure “how well one can predict the coarse category of a fine category given its representational cluster”, and *vice versa*. Mutual information is well-suited to measure this relationship, and we compute it between the set of representational clusters, H, and the set of coarse categories, $C^{\text{coarse}}$, as follows:


$$\begin{array}{l} I\left[H;C^{\text{coarse}}\right]\\ =S\left(C^{\text{coarse}}\right)+\underset{i}{\sum }P\left(H_{i}\right)\underset{j}{\sum }\\ P\left(C_{j}^{\text{coarse}}|H_{i}\right)\log_{2}P\left(C_{j}^{\text{coarse}}|H_{i}\right) \end{array}$$ (10)

Here, *P*(*H*_*i*_) is the proportion of fine categories assigned to representational cluster *H*_*i*_, and $P\left(C_{j}^{\text{coarse}}|H_{i}\right)$ is the conditional probability that a fine category from cluster *H*_*i*_ belongs to the coarse category $C_{j}^{\text{coarse}}$. These probabilities are estimated from the observed frequencies of coarse categories and representational clusters that fine categories belong to (Figure 3C). $S\left(C^{\text{coarse}}\right)$ denotes the entropy of the coarse-category distribution. Since each of the 10 coarse categories contains the same number of fine categories, this distribution is uniform, and its entropy is log_2_(10) ≈ 3.32 bits.

Note that the mutual information estimates computed using this procedure can be highly biased and could be inaccurate (Paninski, 2003; Laparra et al., 2025). In the condition used in this work, the number of samples for estimation (50 fine categories) is much smaller than the number of bins in the joint distribution (10 coarse categories *times* 10 representational clusters = 100 bins). In such sparse sampling regimes, this procedure is expected to provide inaccurate estimates. To further ensure the reliability of effects of pre-training suggested by the results of this evaluation including such naive and potentially biased estimates of mutual information, we also show the values computed for randomly initialized networks to compare against those for the trained models in the Results section. We also conducted a simulation on the extent to which these naive estimates of mutual information could potentially be biased under an assumption of parameterized categorical joint distribution of the coarse object categories and the representational clusters in the model (Supplementary Section 3).

#### 2.5.2 Matrix similarity evaluation: comparison to human confusion matrix on CIFAR-10

In this evaluation, we compare the classification performance of the networks with that of human participants, based on the CIFAR-10H dataset (Battleday et al., 2020) (see Section 2.2.2). This dataset contains results from a behavioral experiment in which human participants were asked to classify images from the CIFAER-10 dataset into 10 object categories. Using these experimental data, we constructed a pseudo-confusion matrix that reflects the “average human perception” for this categorization task.

To evaluate the similarity between the internal representations of the models and human recognition, we computed Spearman's rank correlation between the human pseudo-confusion matrix and the error pattern matrices produced by the networks in the multi-class few-shot learning. This analysis quantifies the correspondence between inter-category similarity as perceived by humans and the representational similarity of categories in the neural networks.

### 2.6 Network architecture

**Figure 4:**
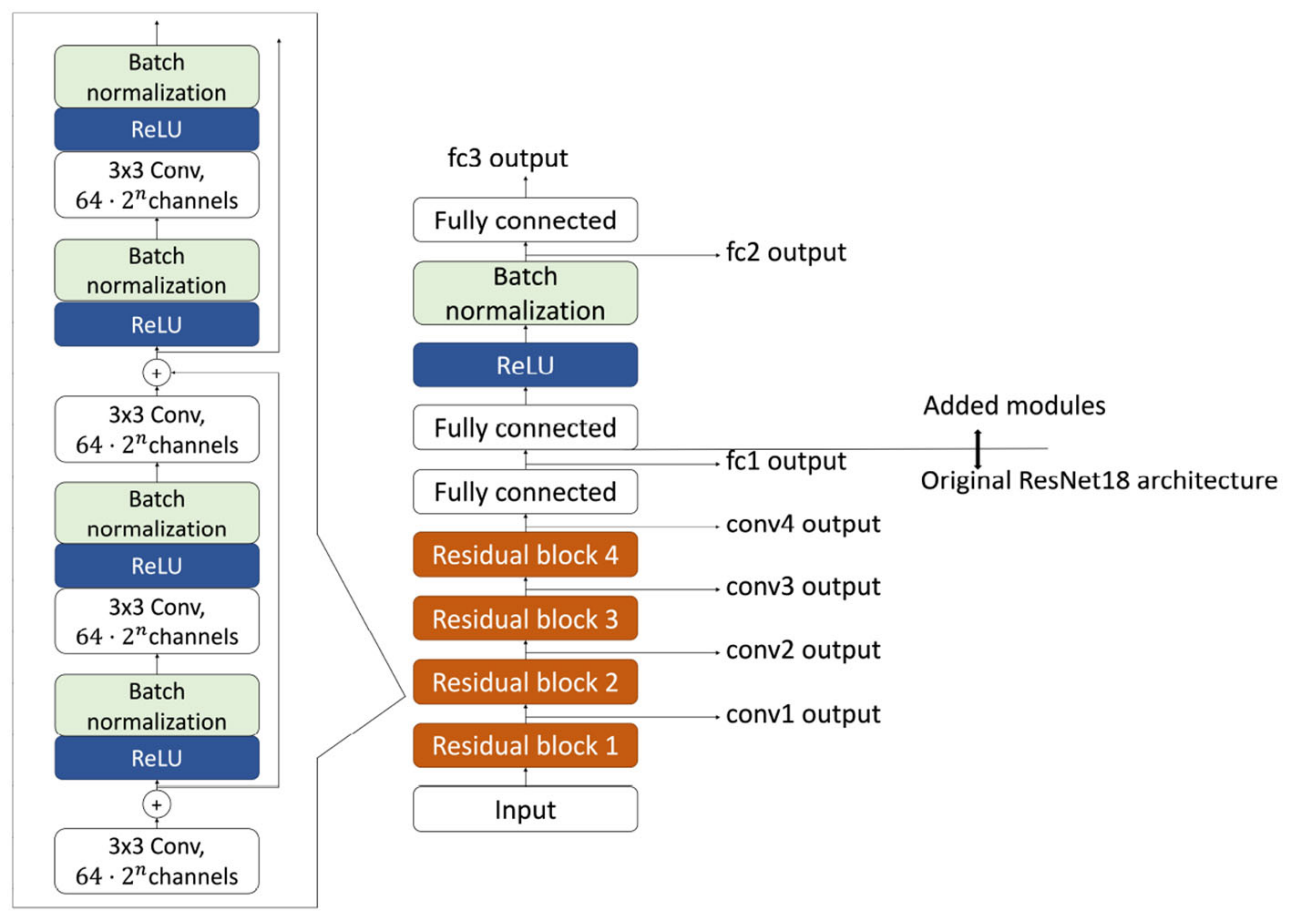
Employed architecture of ResNet-18 network. In addition to the originally proposed architecture, we added additional layers. The objective functions for both contrastive learning and supervised learning are computed in the output of the final layer.

In the present study, we employ a modified ResNet-18 (He et al., 2015) as the encoder backbone for the neural networks. The original ResNet-18 architecture consists of 18 layers, including residual connections. To stabilize the learning process, particularly for SimCLR, we added a fully connected layer followed by a ReLU activation, batch normalization, and a second fully connected layer (Figure 4). Note that in both the supervised and contrastive learning settings, the function *f* appearing in the loss definitions refers to the output of this final additional module. Accordingly, the output dimensionality of the final fully connected layer is set to *d* for contrastive learning and |C| for supervised learning settings.

Overall, the network consists of four residual blocks, each comprising a stack of convolution-normalization modules (Figure 4, left), followed by three fully connected layers with nonlinear transformations. In this article, we refer to the outputs of the residual blocks as “conv*n*” outputs, where conv denotes convolution and the index n increases with network depth (Figure 4, right bottom). The output of the first fully connected layer is referred to as the “fc1” output, where fc denotes fully connected. Likewise, we denote the output of the subsequent batch normalization layer after the second fully connected layer as “fc2”, and the final network output as “fc3” (Figure 4, right top).

## 3 Results

Before presenting the evaluation results, we briefly review the evaluation procedure introduced in Section 2 and Figure 1. To examine whether the DCNNs acquire internal representations of objects resembling human semantics and recognition through a learning objective that does not require explicit supervision over object categories, we first pre-trained the DCNN with self-supervised contrastive learning (Figure 1A, top; Figure 2), using a ResNet18-based architecture (Figure 4). Because this learning framework does not explicitly use supervision over object categories, it does not necessarily guarantee that the learned representations will be organized categorically or align with human semantics and recognition. The learned representations were then evaluated on a downstream pairwise few-shot learning task with novel object categories not included in pre-training (Figure 1A, bottom). The results of this evaluation are presented in Section 3.1. Next, in Section 3.2, we show evaluation of whether the inter-category similarity structure reflects the semantic organization of categories in humans, using hierarchical clustering based on the error pattern matrices obtained from the pairwise few-shot learning evaluation (Figure 1B, Figure 3). Finally, we evaluated the extent to which the object category representations in the trained DCNN resemble the confusion patterns observed in human recognition (Figure 1C). The results of this evaluation are presented in Section 3.3.

### 3.1 Performance on few-shot novel category discrimination

First, we evaluated the performance of a DCNN trained with self-supervised learning (SimCLR) on the task of pairwise few-shot discrimination of novel object categories, and constructed error pattern matrices from the results. As a baseline for comparison, we also evaluated a DCNN trained with supervised learning. In the pre-training phase, both networks were trained on image samples from 50 known object categories out of the 100 pre-defined categories in the CIFAR-100 dataset. After pre-training, we assessed few-shot discrimination performance using image samples from the remaining 50 novel categories.

**Figure 5:**
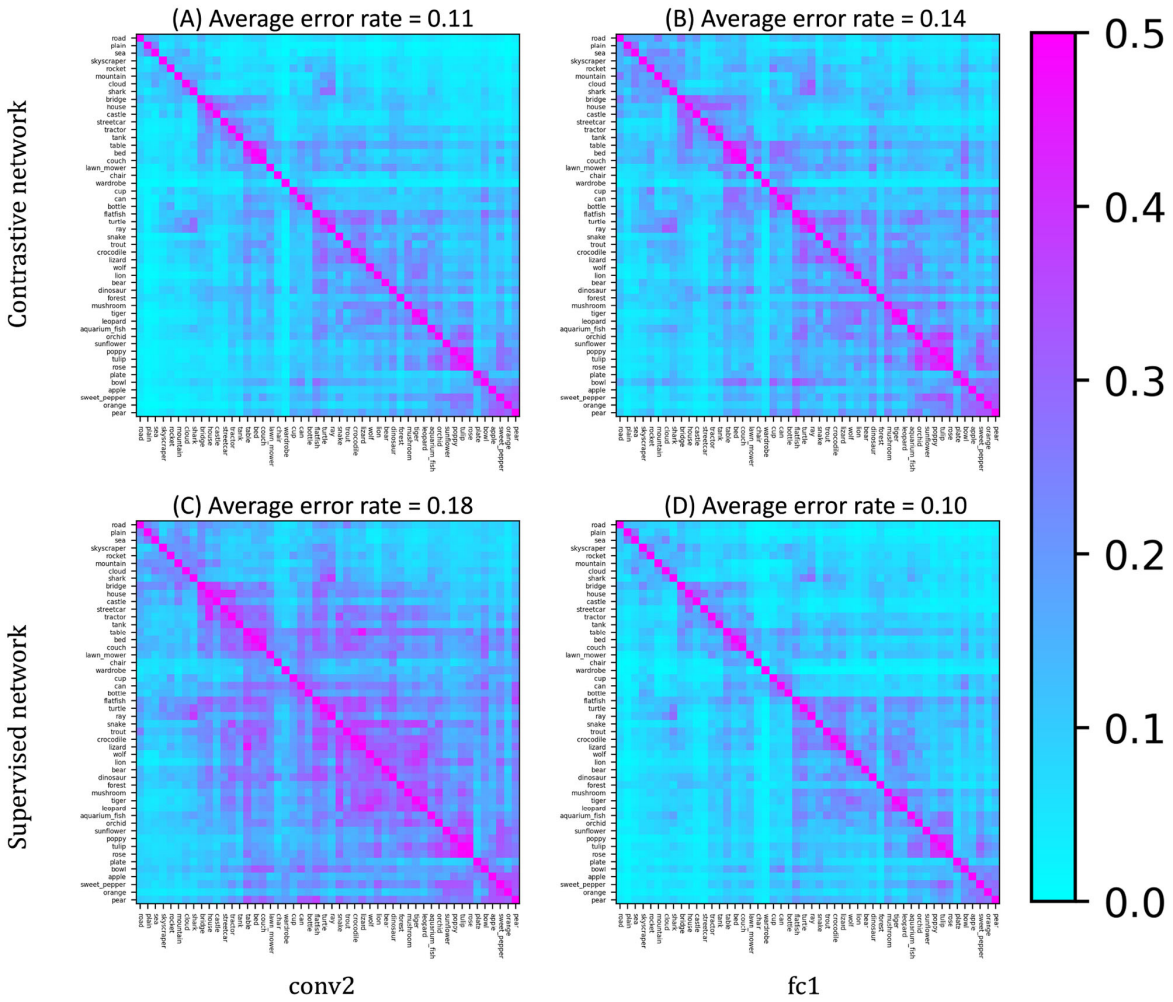
Error pattern matrices of the networks in representative layers. **(A, B)** show the results on the network trained by contrastive learning, and **(C, D)** are the results of the supervised baseline. The panels on the left **(A, C)** and right **(B, D)** show the results from the shallower conv2 layer and deeper fc1 layer, respectively. While the supervised baseline model showed slightly lower accuracy in the shallower layer, the DCNN trained by contrastive learning exhibited accurate discriminations of novel object categories in both layers.

We first present the error pattern matrices for the few-shot discrimination task involving the novel object categories, computed from the internal representations at two layers: convolutional layer 2 (conv2) and fully connected layer 1 (fc1) (Figure 5). These layers are those in which the minimum value of average error rates were achieved in the self-supervised and supervised models, respectively. Each element in the matrix indicates the error rate for discriminating a pair of novel categories, averaged over multiple trials using different few-shot samples. Bluish elements correspond to category pairs with error rates below approximately 15% (i.e., accuracy above 85%), whereas reddish elements indicate near-chance-level performance (~50%). In the DCNN trained with self-supervised contrastive learning, most category pairs were discriminated with accuracy exceeding 80%. The average accuracies in conv2 and fc1 were approximately 89% and 86%, respectively. Although there was a slight difference in performance between the two layers, no drastic degradation or improvement was observed. In contrast, the baseline DCNN trained with supervised learning exhibited a higher accuracy at the deeper fc1 layer (approximately 90%) compared to the shallower conv2 layer. For more detailed results across all layers, see Supplementary Figure S4.

**Figure 6:**
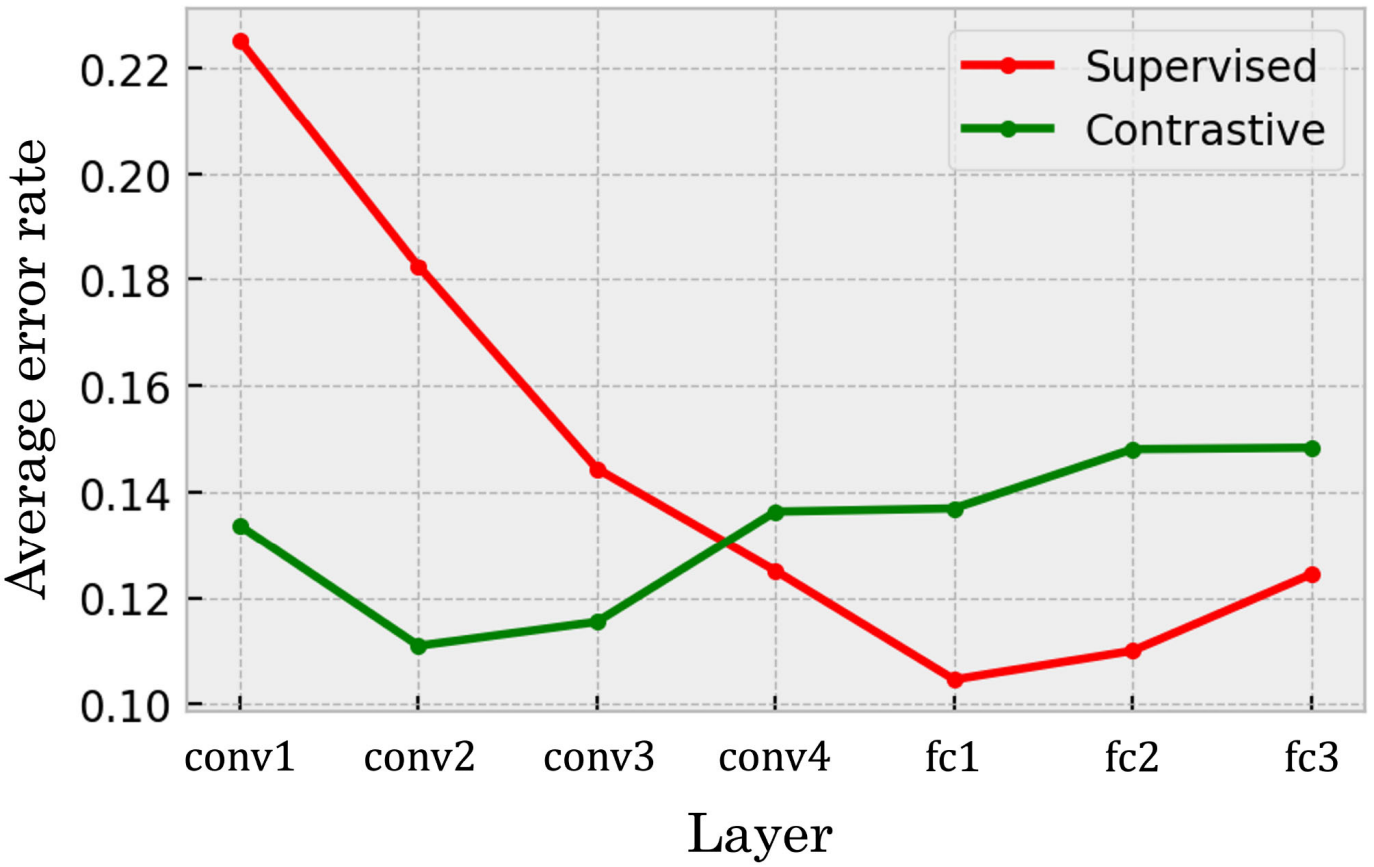
Mean error rate of the networks in different layers on the pairwise few-shot learning task. The green line indicates the error rates in the DCNN trained with self-supervised contrastive learning. In contrast, the red line corresponds to error rates in the supervised baseline. The horizontal axis represents the layer indices. conv layers consist of convolutional processing, while fc layers are fully-connected layers. While the supervised baseline model provided higher accuracy in the deeper layers, the contrastive model also exhibited high accuracy along the hierarchy of the network.

For a more detailed comparison between the self-supervised and supervised models in terms of layer-wise performance differences, we present the average error rates computed across all layers in each model in Figure 6. Each point in the graph represents the mean value of an error pattern matrix, excluding the diagonal elements, calculated from the representations at a specific layer of the network. In the self-supervised model, the accuracy of few-shot novel category discrimination did not show strong dependence on layer depth; performance remained relatively stable across the hierarchy. In contrast, the supervised model exhibited a clear trend: the average error rate was higher in the shallower layers and gradually decreased in the deeper layers. Although the self-supervised model outperformed the supervised model in the shallower layers, both models achieved similar levels of accuracy at their respective best-performing layers. We also evaluated a DCNN trained using SimSiam, another contrastive learning algorithm. The results were qualitatively similar to those of SimCLR, although the overall accuracy of the SimSiam model was lower (see Supplementary Figure S1A).

### 3.2 Correspondence between unsupervised clustering of the DCNN's representations and human semantic object categories

To examine whether the representations in the DCNNs reflect the human-like semantic organization of object categories, we conducted the clustering-based evaluation (Section 2.5.1) to assess the correspondence between the representational similarity structure of novel fine categories in the DCNNs and the semantic relationships among categories defined by humans. The hierarchical relationships between lower- and higher-level object concepts are provided by the fine and coarse categories defined in the CIFAR-100 dataset. The procedure consists of three steps: extracting representational clusters of fine categories, constructing a joint histogram (probability distribution) indicating how fine categories are assigned to each pair of coarse category and representational cluster, and evaluating the mutual information between coarse categories and representational clusters.

**Figure 7:**
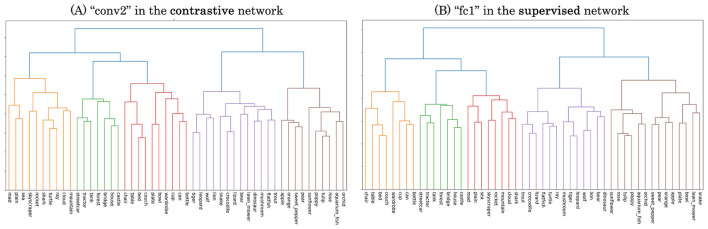
Dendrograms resulting from hierarchical clustering on error pattern matrices of pairwise few-shot learning. **(A)** conv2 layer of the self-supervised network. **(B)** fc1 layer of the supervised network.

First, we extracted *representational clusters* using the error pattern matrices obtained from the pairwise few-shot learning evaluation. These matrices (Figure 5) indicate the error rates in discriminating each pair of novel categories, which can be interpreted as measures of similarity between categories from the perspective of the DCNN representations. We converted the similarity matrices into representational distance matrices by taking their complements (1 − similarity) and performed hierarchical clustering on them. Examples of the resulting dendrograms are shown in Figure 7, derived from the conv2 layer of the self-supervised network (Figure 7A) and the fc1 layer of the supervised network (Figure 7B), which are the layers with the lowest average error rates in pairwise few-shot learning for each model as in Figure 5. For each dendrogram, we identified the dissimilarity level (vertical axis) at which the number of highest-level clusters matched the number of novel coarse categories (10) in the CIFAR-100 dataset. We refer to the clusters obtained at this threshold as the *representational clusters* of the DCNNs.

**Figure 8:**
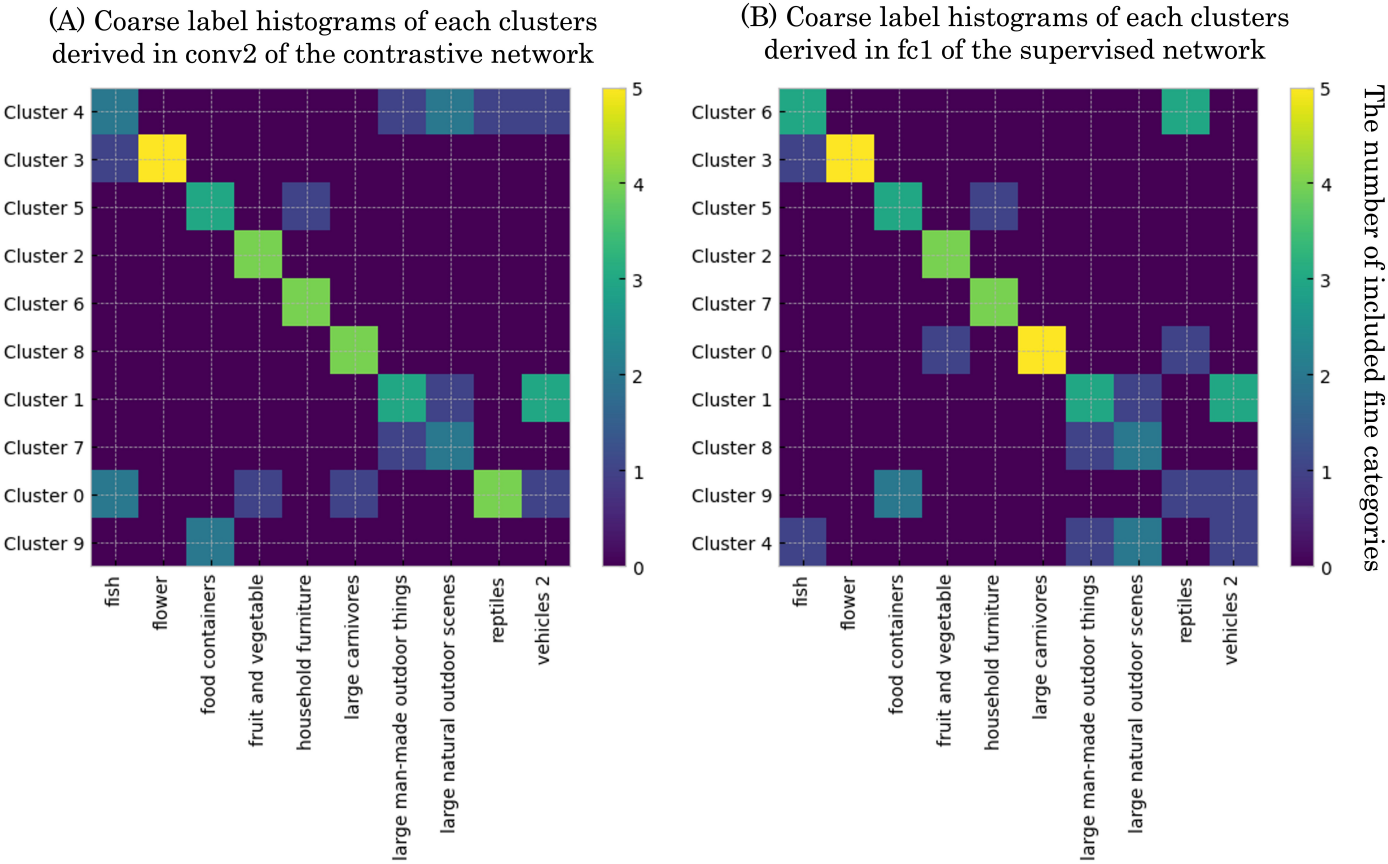
Derived relationships between hierarchical clusters on error pattern matrices of the networks and coarse labels. Rows correspond to clusters, wherein each component is the number of fine categories belonging to a cluster and to a coarse category at the same time. **(A)** conv2 of the self-supervised network. **(B)** fc1 of the supervised network.

Given the representational clusters, we constructed a joint histogram representing the probability that a fine category belongs simultaneously to a coarse category and to one of the representational clusters. Each entry in the histograms shown in Figure 8 indicates the number of fine categories assigned to a particular coarse category (column) and representational cluster (row). Thus, horizontal summation and normalization of a histogram yield *P*(*H*), while extracting a single row corresponds to computing *P*(*C*^coarse^|*H*) in Equation 10. Each matrix entry therefore reflects how many fine-grained categories in a cluster are associated with each coarse category. For detailed layer-wise results, see Supplementary Figure S3.

In the self-supervised model (Figure 8A, conv2), diagonal elements had consistently higher values than off-diagonal ones, suggesting that most clusters were highly aligned with specific coarse categories. Even in clusters with weaker correspondence to coarse categories, the included categories were semantically coherent, for instance, clusters rarely mixed coarse categories like “artifacts” and “natural objects”. These results indicate that the self-supervised DCNN grouped novel categories in ways that are consistent with human semantic similarity. The supervised model showed a broadly similar pattern, except that a clear cluster corresponding to the “reptiles” category, which was observed in the self-supervised model, was missing.

**Figure 9:**
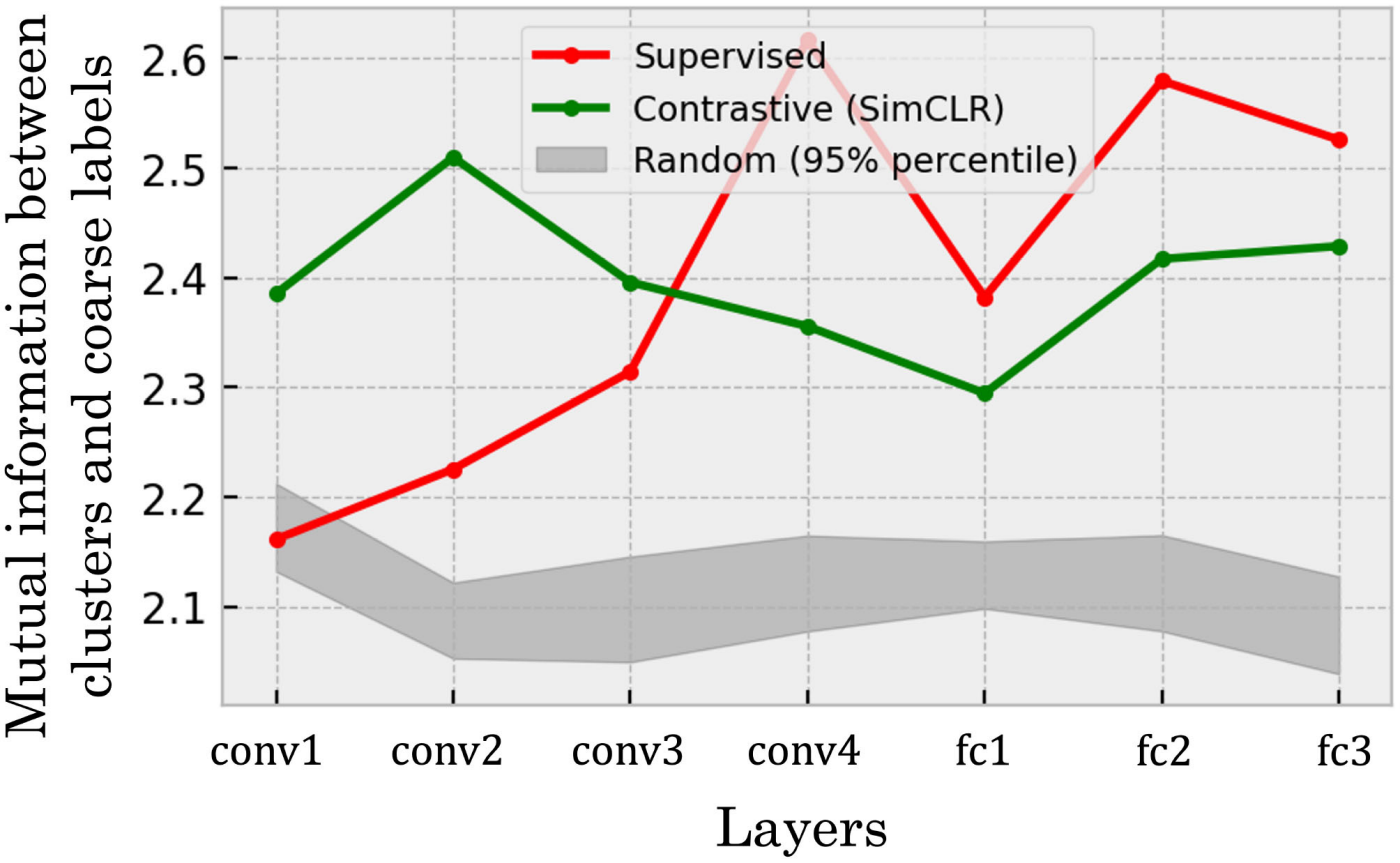
Estimates of mutual information between hierarchical clusters and coarse categories. The green line indicates the results from the self-supervised model, whereas the red line represents the results from the supervised model. The gray band in the figure represents the chance-level mutual information, with 95% percentile interval of the values, computed from 20 randomly initialized models. The results showed that both of the trained models comparably showed mutual information estimates substantially above the chance-level values, implying that the self-supervised learning can develop internal representations of object categories with the structure consistent with the human semantics at the same level as the supervised model. Note the estimated mutual information potentially have biases and variances due to the number of fine categories being smaller than the number of entries in the empirical joint histogram. Hence, the mutual information estimates should be interpreted in terms of the variance associated to similar scenarios (*e.g*., Supplementary Section 3).

To quantify the consistency between representational clusters and human semantics, we computed the potentially biased naive mutual information estimates between clusters and coarse categories in each layer (see Section 2.5.1). Figure 9 shows the layer-wise mutual information estimates for both networks. The green line shows the mutual information in the self-supervised model at each layer, while the red dashed line is that in the supervised model. Here, as mentioned in Section 2.5.1, the procedure of computing the mutual information values shown in Figure 9 includes estimation of the joint distribution in a sparse sampling regime, and such an estimation is often considered positively biased (Paninski, 2003; Laparra et al., 2025). To provide a baseline to be compared to such biased estimates of mutual information, we also show 95% percentile intervals of mutual information estimates computed for 20 randomly initialized neural networks (Figure 9, gray band).

Our primary finding is that the self-supervised network achieves a high structural consistency with human semantics, a level that is not only substantially above the chance-level baseline but also comparable to that of the supervised network. This reference allows us to identify two robust observations. First, the self-supervised model maintains high mutual information estimates consistently across all layers. Second, the supervised model also performs well above chance, with the only exception being its early layers, where performance is close to the chance level before increasing significantly in deeper layers. While these broad patterns are clear, the potential variance of the estimates prevents a more fine-grained comparison, such as definitively concluding which model performs better globally or interpreting the significance of minor layer-to-layer fluctuations.

Overall, our main conclusion is that a self-supervised network can develop internal representations with a categorical structure that is significantly aligned with human semantics, reaching a level of consistency comparable to a supervised model. Conversely, a significant drop of the mutual information estimates in the deeper layers were observed in the SimSiam-trained DCNN (see Supplementary Figure S1B). This implies that the consistency of the representations to human semantics can be dependent on the specific variants of objectives within self-supervised learning.

### 3.3 Similarity between the error patterns of classification in the DCNNs and human behavioral data

Based on the findings from the previous subsections, we next investigated whether the similarity between object categories in DCNNs was consistent with human perception or recognition. To this end, we used the CIFAR-10H dataset (Battleday et al., 2020), which contains behavioral data from human participants performing a 10-class object classification task on the CIFAR-10 dataset. We compared the confusion matrices derived from DCNNs performing multi-class few-shot learning on CIRFAR-10 images with the confusion matrix computed from human responses provided by CIFAR-10H (see Section 2.2.2 for a detailed procedure to compute the human confusion matrix). Similarity was quantified as the Spearman rank correlation coefficient. Note that the CIFAR-10 object categories do not overlap with those in CIFAR-100 used for training the DCNNs, guaranteeing that the object categories in this dataset are also novel to the networks.

**Figure 10:**
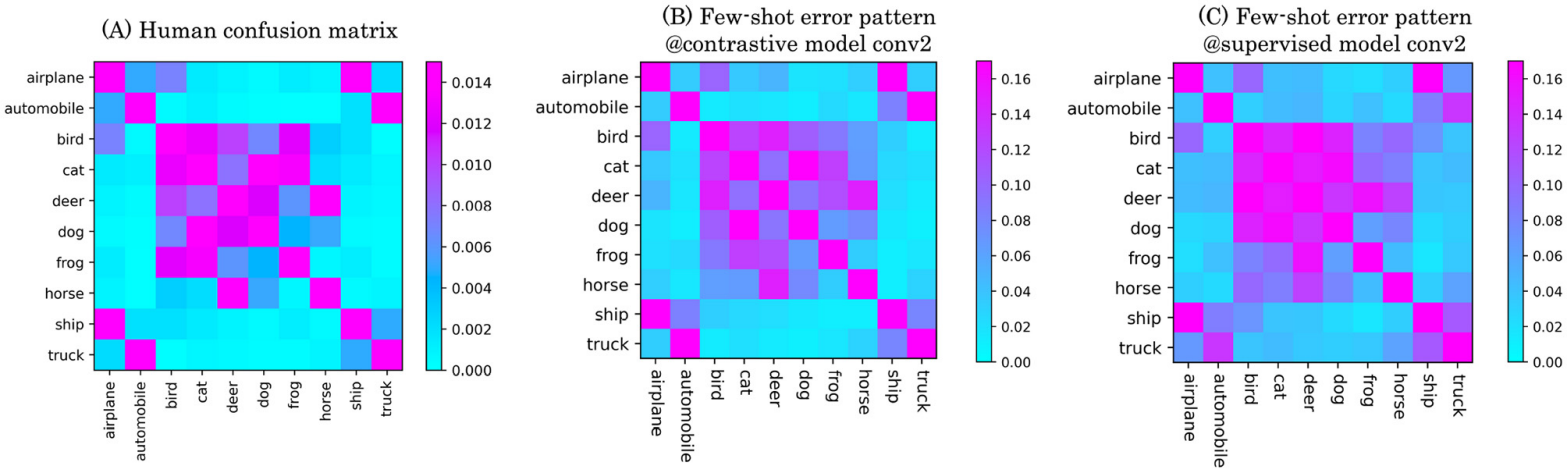
Confusion matrices on 10-class object categorization of the CIFAR-10 dataset. **(A)** Average performance of 2,750 human participants. **(B)** Confusion matrix of the contrastive model. **(C)** Confusion matrix of the supervised model. In shallower layers, we observed a higher similarity to human error patterns in the contrastive model.

The confusion matrices computed from the self-supervised and supervised DCNNs showed qualitatively similar pattern of confusion to the human recognition. Figure 10 (left) shows the confusion matrix generated from human behavioral data. As adult participants generally performed this task with high accuracy, the resulting confusion matrix exhibits relatively small error values. When comparing this to the confusion matrix of the self-supervised model (Figure 10, middle), we observe similar global patterns. In particular, characteristic confusion structures present around the center and at the four corners of both matrices. These patterns suggest that object category pairs which are difficult for humans to perceptually discriminate are also similarly represented in the self-supervised model. The supervised baseline model (Figure 10, right) also produced a confusion matrix resembling the human pattern, but with generally higher error rates. This increase in errors obscured finer details in the confusion structure, resulting in a weaker alignment with both the human and self-supervised model matrices.

**Figure 11:**
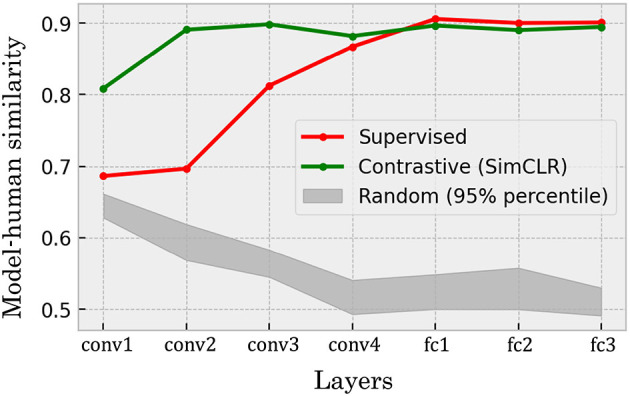
The Spearman rank correlations between models' confusion matrices and that of human participants computed from CIFAR-10H annotations. The self-supervised (green line) DCNN exhibited high values in both shallower and deeper layers, while the supervised model (red line) showed lower similarity to human behavioral data in the shallower layers. The gray band shows 95% percentile interval of the same quantities computed for 20 randomly initialized networks. The random networks exhibited an opposing trend to the trained networks in which the deeper layers shows relatively lower correlation coefficients.

A precise evaluation of the similarity between the categorical relationship structures in the DCNNs' representations and human recognition revealed high similarities of the representations in the self-supervised network to human recognition throughout the network hierarchy, the supervised model showed similarities that increase particularly in the deeper layers (Figure 11). In the self-supervised model (Figure 11, green line), correlation coefficients ranged from approximately 0.8 to 0.9 across the network hierarchy. This indicates that the model trained by the self-supervised learning acquires the representations of visual objects that strongly and stably align with humans' perceptual similarities between the categories of them. In contrast, the supervised model (Figure 11, red line) showed lower correlations in its early layers. The correlation coefficients increased along the network hierarchy, to the same level as the self-supervised model in the layers deeper than conv3.

Similarly to the quantification of the similarity of fine-coarse categorical inclusion between the models and humans (Section 3.2), the Spearman rank correlations are also potentially biased. In this particular evaluation, the bias is considered to be the inductive bias induced by the convolution-based network architecture. To clarify the relevance of the quantitative evaluations under the potential biases, we also showed the 95% percentile intervals of the results of the same analyses performed on 20 randomly initialized networks with the same architecture (Figure 11, gray band).

The trend observed in the random networks were different from the self-supervised and supervised models. First, the overall correlation coefficients were lower (in an approximate range between 0.5 to 0.65) than the levels of the self-supervised (0.8–0.9) and the supervised (0.7 to 0.9) models. In addition, the trend of the values were opposite; while the trained models tend to show higher correlation coefficients in the deeper layers, the random networks showed lower correlation values in those layers.

Overall, the results suggest that both the self-supervised and supervised models obtained the representations of object categories resembling the similarity structure in human recognition. In particular, both models constantly showed the correlation level above that of the random networks. Furthermore, the trends of the correlations were opposite between the random and trained networks; the deeper layers of the random networks showed decreasing correlation, while those of the trained networks exhibited increasing correlation coefficients. These opposing trends further clarify that the trained models have higher similarity of inter-categorical confusion structure to human recognition especially in the deeper layers.

## 4 Discussion

In this study, we investigated whether deep convolutional neural networks (DCNNs) trained with self-supervised learning could acquire internal representations that resemble those of human semantic understanding and perceptual recognition. To this end, we evaluated the networks' performance on few-shot learning tasks involving novel object categories.

Our findings revealed three main results. First, internal representations learned through self-supervised contrastive learning (1) enabled accurate few-shot classification of novel object categories. Second, these representations (2) exhibited inter-categorical structures that closely mirrored human semantic organization. Third, they (3) produced error patterns in few-shot classification tasks that were similar to those observed in human object recognition.

### 4.1 Internal representations of self-supervised learning

Here, we discuss the non-trivial aspects of the internal representations obtained through self-supervised learning. Our findings that self-supervised contrastive learning can yield internal representations enabling accurate few-shot classification, and that the inter-categorical structure of these representations aligns with human semantic and perceptual recognitions are far from obvious.

This is because contrastive learning, particularly in the SimCLR framework, is designed to pull together positive pairs and push apart negative pairs, without any access to object category labels (as reflected in the objective function; Equations 1, 2). Therefore, there is no explicit reason why such training should result in representations that are both categorical and aligned with human semantics. In fact, the emergence of categorical structure through self-supervised learning may appear even more non-trivial than in supervised learning, where explicit category information is provided and thus encourages such structure. It is worth noting, however, that even in supervised learning, the acquisition of categorical representations for novel, unseen categories is not guaranteed or trivial (Sorscher et al., 2022).

Furthermore, our comparisons between DCNNs trained via self-supervised and supervised learning revealed additional non-trivial findings. Across both few-shot learning performance and correspondence to human perception, we observed qualitative similarities (e.g., error patterns in Figures 5, 10; clustering structures in Figures 7, 8) as well as quantitative ones (e.g., mean error rates in Figure 6, mutual information in Figure 9, and rank correlations with human confusion matrices in Figure 11).

Given the substantial difference in training objectives between self-supervised and supervised learning, these converging results are highly non-trivial and suggest a remarkable similarity in the internal representations learned by both approaches. While several theoretical connections between supervised and self-supervised objectives have been proposed (Arora et al., 2019; Bao et al., 2022; Nozawa and Sato, 2021), there is currently no comprehensive theoretical explanation for the observed alignment in representations between models trained with these distinct objectives. Further theoretical investigation is needed to clarify why and how such similarities emerge between contrastive and supervised learning.

These insights raise the possibility that aspects of human semantic understanding may emerge in the absence of explicit external supervision. This idea naturally leads to the discussion in Subsection 4.2, where we explore the implications of these findings for language acquisition and development.

### 4.2 Formation of semantics in humans

Although we focused on the visual processing in the DCNNs and their internal representations of images and objects acquired through different learning mechanisms, the results can be interpreted in relation to the structure of human language. Specifically, our pairwise few-shot learning evaluation in Section 3.1 was conducted using category labels from the CIFAR-100 dataset, which are based on English vocabulary. From this perspective, the experiment can be interpreted as a test of whether visual object categories referred to by different English terms are linearly separable within the network's internal representation. Our results demonstrated that the self-supervised DCNN performed few-shot learning successfully, implying that the internal representations contain categorical structures aligned with human linguistic categorization.

Additionally, the comparison of the clusters in the networks' internal representations with coarse-grained category labels (Section 3.2) also provided an implication for understanding the human languages. These coarse categories used in the investigation, also derived from the English language, were found to correspond well with the clusters formed in the DCNN's internal representations. This again suggests a correspondence between the structure of language-based categories and the internal representations formed through self-supervised learning.

Based on these findings, we speculate that the categorical structure of language might, at least in part, emerge from the separability of object representations in the brain–representations that may be shaped through self-supervised learning. While the precise structure of these representations can vary depending on the learning environment and input statistics, it is plausible that self-supervised learning yields common, structured representations across individuals, which in turn inform the emergence of linguistic categories.

Conversely, the reverse direction of influence, where language shapes perceptual recognition and even neural representation, has also been widely discussed. A prominent example is the Sapir-Whorf hypothesis (Whorf, 2012; Brutyan, 1969; Kay and Kempton, 1984), which posits that the structure of language can shape and even constrain cognitive perception. For instance, the conflation of “butterflies” and “moths” under the single French term *papillon* may, under this hypothesis, blur perceptual distinctions for native French speakers. Empirical studies have shown that native speakers of different languages may differ in their perception of objects, time, color, and other aspects of experience that are linguistically encoded (Boroditsky, 2001; Lupyan et al., 2020). Although we did not directly address this reverse effect in our study, it remains an important direction for understanding how language influences the development of neural representations.

Taking both directions into account, it seems reasonable to hypothesize that neural representations are initially formed through self-supervised learning during early development such as infancy, and subsequently fine-tuned by language-based supervision. Most computational studies to date have focused on the outcome of a single learning rule. To better understand brain-like learning mechanisms, future research should consider how the interplay between self-supervised learning and supervised fine-tuning models the developmental progression of neural representations from infancy to adulthood.

### 4.3 Toward a more biologically plausible learning mechanism

Here, we discuss the implications of our findings for understanding the formation of categorical representations in biological neural systems. The central result of this study is that a DCNN trained with self-supervised contrastive learning can develop internal representations of visual objects closely resembling human perceptual recognition and semantic organization. If a similar mechanism operates in biological brains, abstract categorical representations might be naturally formed prior to language-based learning. Below, we first address how contrastive learning might be biologically implemented through prediction-based learning mechanisms and then discuss how biologically plausible visual input augmentations naturally arise from such mechanisms.

A plausible implementation of contrastive learning in biological brains would be prediction-based learning, a central component in many theoretical neural processing frameworks (Rao and Ballard, 1999; Friston, 2010). Prior studies have formalized contrastive learning using predictive paradigms by defining temporally proximal events as positive pairs and distant events as negative pairs (van den Oord et al., 2018; Lowe et al., 2019; Illing et al., 2020). Unlike SimCLR, which explicitly contrasts positive and negative pairs within the same batch, prediction-based learning naturally distinguishes positive and negative pairs through temporal proximity without explicit negative sampling or specific architectural constraints. Despite these differences, both SimCLR and biologically plausible prediction-based learning fundamentally share the principle of forming structured representations by comparing related and unrelated experiences, highlighting the biological relevance of the computational principles demonstrated by SimCLR in our study.

Furthermore, if we regard prediction-based learning as a plausible candidate, the visual input augmentations integral to contrastive learning such as image rotations or random cropping can naturally occur through bodily movements and sensorimotor interactions, in addition to natural temporal changes in the input from the external environment. Although the artificial augmentations used in this study include those that might not exactly correspond to natural conditions (color distortion, grayscaling, or random blurring), the remaining transformations commonly occur in biological contexts through movements such as head rotations, locomotion, and saccadic eye movements. For instance, neck rotations cause corresponding rotations in retinal images, and moving closer to an object results in a visual effect analogous to cropping. Thus, sensorimotor experiences encountered in early development inherently provide the biological basis for visual augmentations that parallel those used in computational contrastive learning.

Taken together, our finding that abstract, human-like categorical representations can emerge from self-supervised contrastive learning provides a promising basis for understanding how such representations may form in the human brain without explicit supervision. If biologically plausible learning mechanisms—such as prediction-based learning shaped by natural sensorimotor experience—can approximate contrastive learning, as discussed above, then our results suggest that conceptual representations could arise through self-supervised processes alone. Rather than claiming that the brain implements contrastive learning per se, our study identifies a representational target and computational principle that future biologically grounded models can aim to approximate. This offers a concrete step toward linking the unsupervised emergence of conceptual structure in artificial systems to that in biological neural systems.

## Data Availability

The original contributions presented in the study are included in the article/Supplementary material, further inquiries can be directed to the corresponding author.
