# Supplementary material for: Exploring internal representations of self-supervised networks: few-shot learning abilities and comparison with human semantics and recognition of objects
Source: Front Comput Neurosci. 2025 Nov 21;19:1613291. doi: 10.3389/fncom.2025.1613291 (PMC12679296; doi:10.3389/fncom.2025.1613291)
Supplement: Supplementary file 1 [file Data_Sheet_1.pdf]

Supplementary Material

1 SIMSIAM CONTRASTIVE LEARNING

In this section, we introduce SimSiam (Chen *et al.*, 2020) as another example of self-supervised contrastive learning algorithm. By conducting the same series of experiments using another DCNN trained by SimSiam learning algorithm, we can discuss generality of the results reviewed in the main content among different self-supervised contrastive learning algorithms.

The DCNN utilized for SimSiam takes the same encoder architecture as the SimCLR model and the supervised baseline in the main content. The netwrok f consists of a ResNet-18 encoder g and additional NLP (fully connected layers) modules proj.

A characteristic of SimSiam that is different from SimCLR is the loss function and the training procedure. While the network takes a positive sample x^+ and negative samples $\{x_k^-\}_k$ for each anchor input $\tilde{x}$ in SimCLR, SimSiam only takes a positive sample for each anchor. Let us take an anchor input $\tilde{x}$ and its positive sample x^+. For one input, the network computes the representation as the output of g, while the representation is computed as the output of proj for the other. The objective function is the negative cosine similarity between the two representations:

$$l_{\text{siam}}(x, x^+) = -\left\langle \frac{f(x)}{\|f(x)\|_2}, \frac{g(x^+)}{\|g(x^+)\|_2} \right\rangle. \tag{S1}$$

While this metric is asymmetrical with respect to x and x^+, the qualitative role of them can be considered symmetrical: if the positive sample x^+ is treated as an anchor sample, then the anchor sample x can also function as a positive sample. Hence, the full expression of the loss function is the average symmetrized negative cosine similarity:

$$\mathcal{L}_{\text{siam}} = -\mathbb{E}_{x\sim\rho_{\text{im}}, a_1\sim\rho_{\text{aug}}, a_2\sim\rho_{\text{aug}}}\left[\frac{l_{\text{siam}}(a_1(x), a_2(x)) + l_{\text{siam}}(a_2(x), a_1(x))}{2}\right]. \tag{S2}$$

The optimization algorithm for SimSiam is also different from SimCLR. The algorithm is based on error back-propagation. However, the gradient of the loss function is computed only with respect to the first argument of l_{siam}. The gradient used for updates of the synaptic parameters in the DCNNs is then

$$\mathrm{grad}(l_{\text{siam}}(x_1, x_2)) = \frac{\partial}{\partial x_1} l_{\text{siam}}(x_1, x_2). \tag{S3}$$

As mentioned above, SimSiam does not require negative samples for training. This feature reduces the computational complexity of the training procedure. Furthermore, this negative sampling-free learning framework is considered more plausible in biological systems. While SimCLR requires the memory of representations of large amount of negative samples, for SimSiam, the network does not need to store such information and only compares representations at different layers. Although the gradient stopping is still implausible, we assume the relationship of contrastive learning to prediction-based learning mechanisms discussed in the main content also holds for SimSiam.

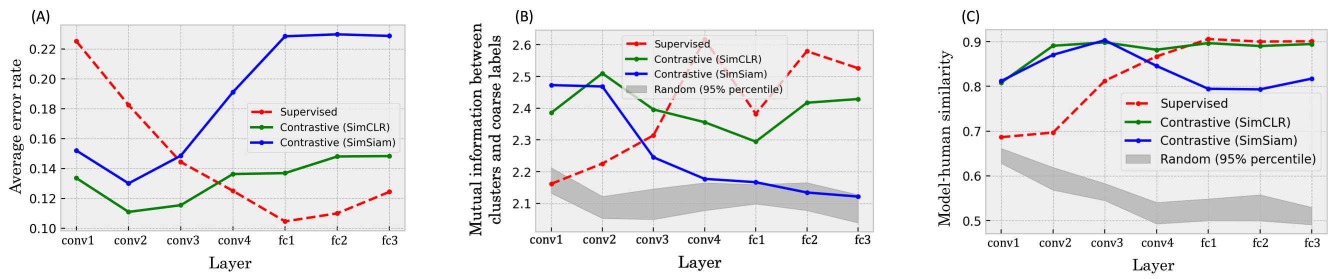

Figure S1. Evaluations of internal representations of the SimSiam model. (A) The layer-wise average error rates of pairwise few-shot learning. All the three subfigures are based on Fig 6, 9, and 11 in the main text. An additional result from the DCNN trained by SimSiam (the blue solid line) is added to each of the subfigures. (B) The layer-wise mutual information between hierarchical clusters of the internal representations and human-defined coarse graining of novel object categories. (C) The layer-wise rank correlation between confusion matrices of multi-class few-shot learning and of human participants.

2 EVALUATIONS OF INTERNAL REPRESENTATIONS OF SIMSIAM MODEL

Here we show the evaluations of a DCNN trained by SimSiam for few-shot learning accuracy (Fig S1, A), correspondences of representations to human semantics (Fig S1, B), and correspondences of representations to recognition (Fig S1, C). The three panels (A), (B), and (C) in Fig S1 respectively correspond to Figs 6, 9, and 11 in the main text. We show the results from the SimSiam model as the blue solid line and also show the results from the supervised model and the SimCLR model as the red dotted line and the green solid line for comparison.

The measured accuracies and correspondences to human semantics / recognition were close to that of the DCNN trained by SimCLR in the shallower layers, while they exhibited lower values in the deeper layers (Figs. S1, A, B, and C). These differences between the two DCNNs trained by SimSiam and SimCLR, especially in the deeper layers, were probably caused by the different learning algorithms. Even with the SimSiam learning method, if the size of the training dataset is much larger than that used in this study, the performance of SimSiam would change and become closer to that of SimCLR, as shown in the previous study (Chen and He, 2020). We leave this issue as a future work.

3 ON THE BIAS IN THE NAIVE ESTIMATION OF MUTUAL INFORMATION

In this work, we conducted a naive estimation of mutual information between representational clusters computed from the internal representations of the trained models and coarse-fine categorical inclusions pre-defined in CIFAR-100 dataset. The bias is primarily due to the imbalance between the number of bins (10 representational clusters $\times$ 10 coarse categories = 100) and the number of fine categories being samples, which is 50 in this work. Since the assignment of the 50 fine categories does not cover the 100 entries in the joint histogram, the computed mutual information is expected to include strong biases. In the main text, we added a presentation of the baseline range of the same naive estimates of mutual information achievable by multiple untrained, randomly initialized networks. We showed that the mutual information estimates for the trained models were substantially higher than the 95% upper percentile. We consider that this result is supportive for our discussion that the coarse-fine inclusion relationships between object categories can naturally emerge in the internal representations of the trained models.

To acknowledge the magnitude of the potential biases from another perspective, here we introduce a simulation that we conducted assuming a parameterized categorical distribution. In this analysis, we

attempt to show the magnitude of possible biases induced to the naive estimation of mutual information. The joint categorical distribution we assume for this simulation is as follows:

$$\begin{aligned} P(X = x_i) &= \frac{1}{10} \text{ for } i = 1, 2, ..., 10 \\ P(Y = y_i | X = x_i) &= \rho \\ P(Y = y_j | X = x_i) &= \frac{1-\rho}{9} \text{ where } i \neq j \end{aligned}$$

In brief, we assume a categorical distribution parametrized by a 1-dimensional parameter ρ which regulates correlation between the variables X and Y. Here, X corresponds to coarse categories defined by human semantics and provided by CIFAR-100 dataset, while Y corresponds to the indices of the representational clusters computed from a neural network. The uniformity of the prior distribution $P(X)$ reflects the fact that the number of fine categories belonging to each coarse category is equal to others (in the case of CIFAR-100 dataset and the evaluation in the main text, the specific number is 5). When ρ is large, it is more probable that Y aligns well with X, which corresponds to the case where each representational cluster contains almost only fine categories belonging to a specific coarse category exclusively. Conversely, when ρ is low at the level near $\rho = 1/10$, this indicates the case where X and Y do not correlate, where the true mutual information is 0.

Under this setting, we show the magnitude of induced biases and variances to the naively estimated mutual information by comparing them to the true values. In particular, for each value of ρ, we show the true value of mutual information and estimated values for varying sample sizes (the number of fine categories) from 25 to 250 with step size being 25. By doing this, we show the dependency of the bias on the naive mutual information estimates on the sample size and the actual correlation between X and Y. The variances are computed from 10,000 random achievements of empirical joint histograms. The true value of the mutual information between X and Y following the joint distribution introduced above can be analytically evaluated:

$$I(X;Y) = \log_2 10 + \rho \log_2 \rho + (1-\rho) \log_2 \left(\frac{1-\rho}{9} \right).$$

The result of the simulation shown in Fig. S2 showed that the naive estimates of mutual information are less biased for larger sample sizes, and for higher ρ values (which leads to higher true mutual information). The difference of biases induced by different values of ρ is most remarkable in the case where the sample size is 25. In this case, the relative values of average mutual information estimates swap from the actual mutual information, and the variances are also extremely large. In the case of 50 samples (highlighted by red dashed box in Fig. S2), which is the same as the evaluation we presented in the main text, such swaps of average values seem alleviated. However, compared to the cases with larger sample sizes, the variances are still large and it is implied that the estimated mutual information values can overlap between different parameters of the joint distribution.

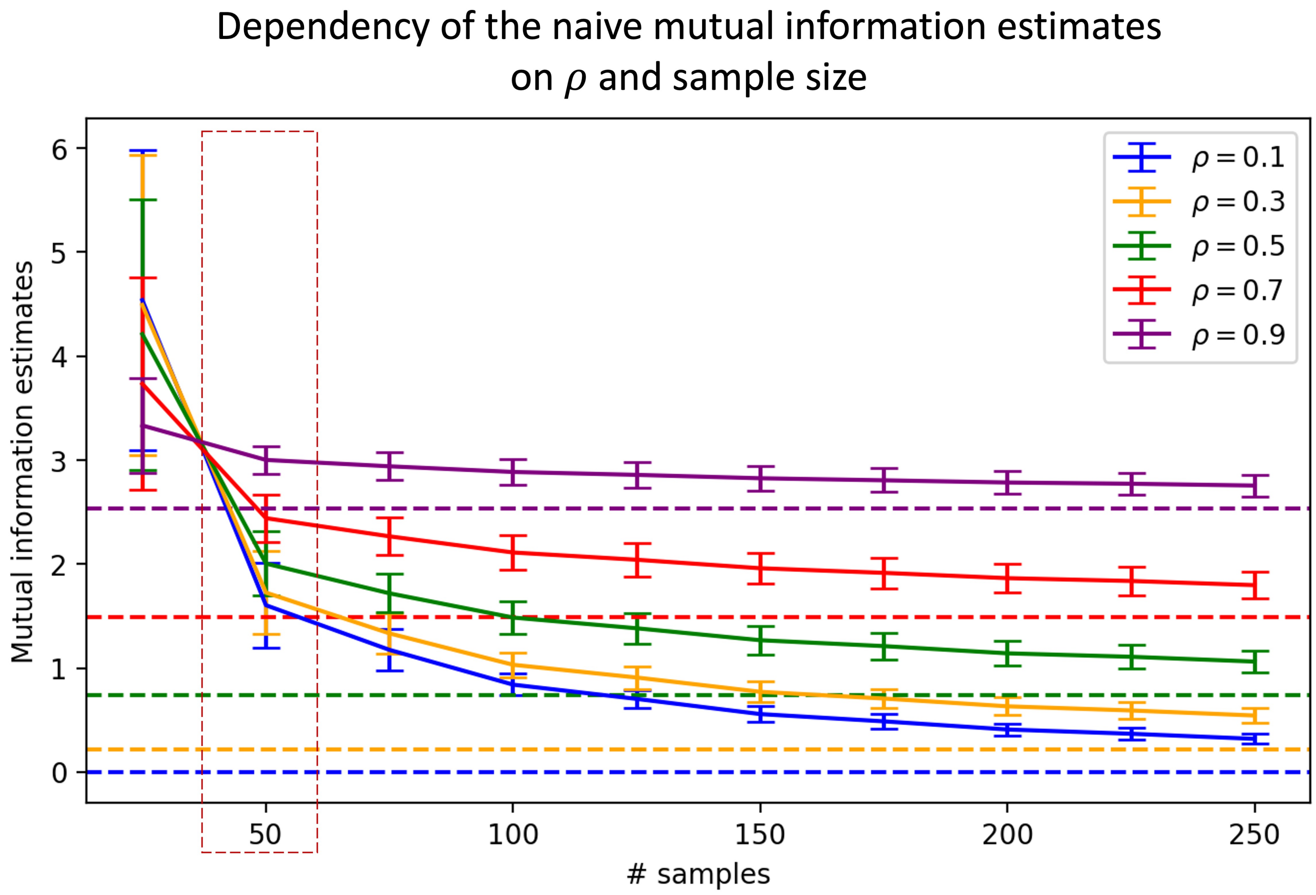

Figure S2. Naive estimates of mutual information for different sparsity of sampling under the virtual distribution setting. The horizontal axis indicates sample size to construct empirical histogram of frequencies of coarse categories and representational clusters. A line with each color corresponds to a value of ρ, where the solid lines are the mutual information estimates and the dashed lines are true mutual information values theoretically calculated from the virtual distribution. The varying magnitudes of mutual information depending on both the sample size and the value of ρ is illustrated.

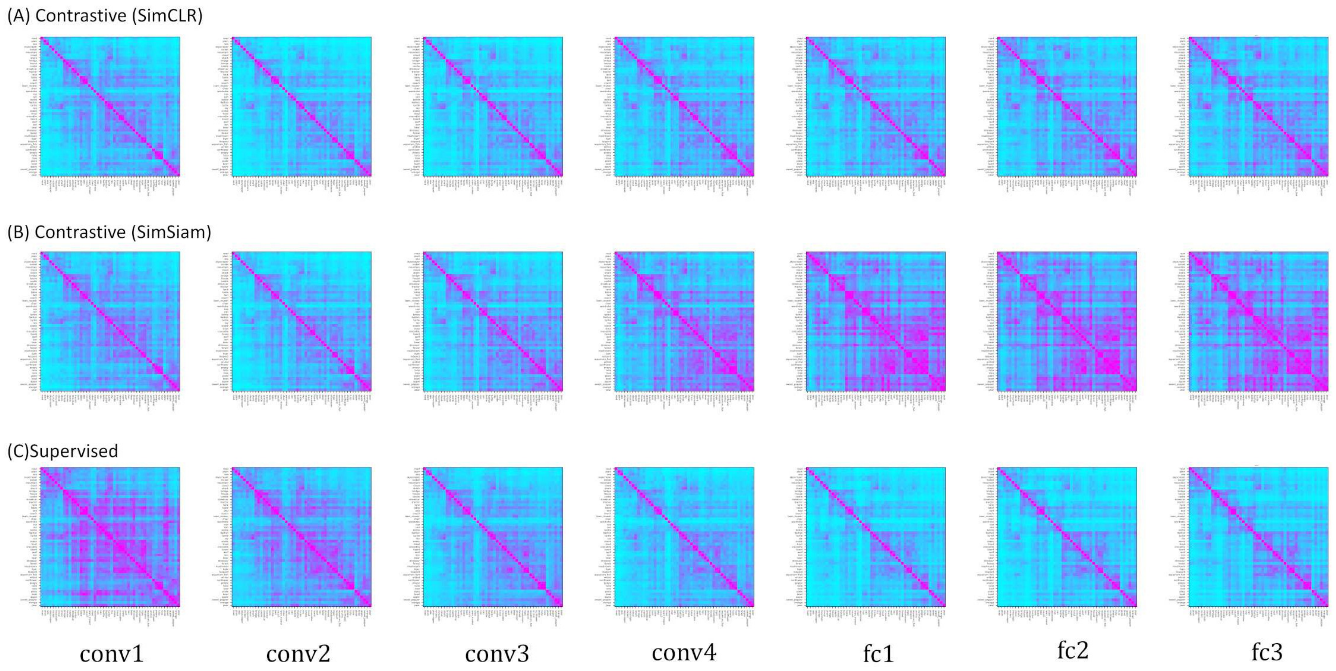

Figure S3. Error pattern matrices of pair-wise few-shot learning of internal representations from all layers of the (A) SimCLR model, (B) SimSiam model, and (C) supervised baseline. Fig S1(A) is obtained by averaging these matrices except for the diagonal elements. While the values vary between the models, the overall error pattern structures were qualitatively similar.

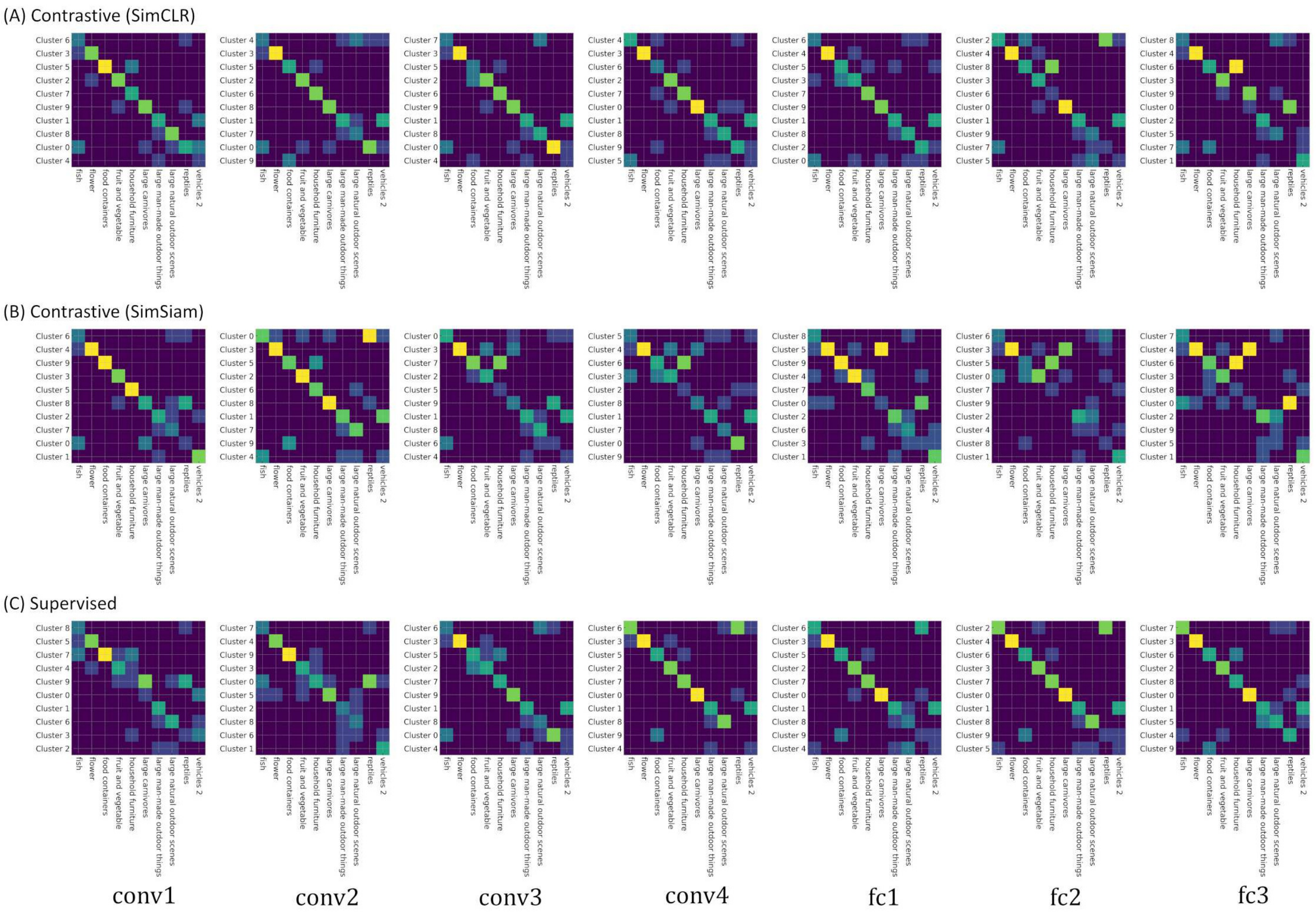

Figure S4. Correspondence of human semantic coarse categories and the clusters of internal representations from all layers of the (A) SimCLR model, (B) SimSiam model, and (C) supervised baseline. Shared tendency between the two models trained by contrastive learning algorithms is that the correspondences between the coarse object categories defined based on human semantics and the hierarchical clusters collapse as it proceeds to the deeper layers, while the supervised baseline shows the opposite tendency. Fig S1(B) can be obtained by calculating mutual information between the sets of coarse categories and hierarchical clusters from these matrices.
